# Comparison of pathogenic bacteria in the upper and lower respiratory tracts of cattle either directly transported to a feedlot or co-mingled at auction markets prior to feedlot placement

**DOI:** 10.3389/fvets.2022.1026470

**Published:** 2023-01-24

**Authors:** Christina Hirsch, Edouard Timsit, Muhammed Salah Uddin, Le Luo Guan, Trevor W. Alexander

**Affiliations:** ^1^Ceva Santé Animale, Libourne, France; ^2^Lethbridge Research and Development Centre, Agriculture and Agri-Food Canada, Lethbridge, AB, Canada; ^3^Department of Agricultural, Food and Nutritional Science, University of Alberta, Edmonton, AB, Canada

**Keywords:** antimicrobial resistance, auction market, bacterial bronchopneumonia, bovine respiratory disease, cattle, feedlot

## Abstract

**Introduction:**

Bacterial bronchopneumonia (BP) has been associated with purchasing cattle through auction markets. However, whether auction markets are a source of BP-associated bacterial pathogens is unknown. This study evaluated prevalence, antimicrobial susceptibility, and genetic relatedness (using pulsed-field gel electrophoresis, PFGE) of *Mannheimia haemolytica, Pasteurella multocida*, and *Histophilus somni* isolated from cattle either transported to an auction market prior to feedlot placement (AUC), or directly to a feedlot from a farm (RANC).

**Methods:**

Two groups of cattle were enrolled (N = 30 per group) from two separate farms with 15 animals from an individual farm designated as AUC or RANC. Deep nasal swab (DNS) and trans-tracheal aspirates (TTA) were collected on day 0 at weaning (T0) and on day 2 at on-arrival processing at the feedlot (T1). The DNS were also collected on day 9 (T2) and day 30 (T3) after arrival at the feedlot.

**Results and discussion:**

In both TTA and DNS, prevalence of bacteria did not differ between AUC and RANC groups (*P* > 0.05). None of the bacteria isolated at T0 were resistant to antimicrobials and diversity of all bacteria was greatest at T0 and T1. In Group 1 cattle, 100% of *P. multocida* isolated at T2 and T3 were multi-drug resistant. These isolates were highly related (>90%) according to PFGE, with most being clones. Though limited in size, results for animals evaluated in this study suggested that auction markets were not a major source of resistant BP pathogens, however, horizontal transmission of a multi-resistant strain of *P. multocida* occurred in a feedlot. Spread of resistant *P. multocida* was likely due to the selective pressures imposed by feedlot antimicrobial use and encoded resistance by the bacteria.

## 1. Introduction

Bacterial bronchopneumonia (BP) remains a challenging health issue facing the North American feedlot industry, causing significant economic losses [over US $500 million annually (1)]. It affects 16.2% of cattle placed in North American feedlots, with ~4% of feedlot cattle dying from this condition (2). The main bacterial pathogens associated with BP are *Mannheimia haemolytica, Pasteurella multocida, Histophilus somni*, and *Mycoplasma bovis*. Although BP is ultimately caused by bacterial pathogens, it is considered a multifactorial disease triggered by a combination of predisposing factors (3, 4). Bacterial pathogens causing BP typically reside in the upper respiratory tract of healthy cattle as commensals (5). However, under specific conditions stressors can suppress host immunity and facilitate proliferation of bacterial pathogens in the upper respiratory tract (URT), followed by colonization and potential infection of the lower respiratory tract (LRT) (6, 7). This explains why beef cattle mostly develop BP during the first 50 days after feedlot placement, as they are exposed to various respiratory viruses (bovine herpes virus 1, bovine respiratory syncytial virus, bovine para-influenza 3 virus, bovine viral diarrhea virus, etc.) at a time when stressors [such as weaning, transportation, co-mingling, adaptation to new diet, surgical procedures (e.g., castration and dehorning)] negatively affect their immune defenses (6, 8).

Trading cattle through auction markets is part of an efficient marketing system in the North American feedlot industry and represents 60% of cattle purchases in Western Canada (9). There is evidence to suggest that cattle purchased through auction markets are at higher risk of developing BP early in the feeding period than cattle coming directly from other beef operations (6). For example, Step et al. (10) reported 42% morbidity related to BP during the first 42 days after arrival in feedlot cattle purchased through auction markets vs. only 11% BP morbidity in ranch-direct cattle. The reasons for this are unclear but may partially be related to auction market cattle being exposed to a greater density of pathogens due to co-mingling and thus an increased risk of pathogen transmission and colonization (11). Furthermore, auction market cattle are exposed to additional stressors (e.g., handling, transportation, co-mingling, and dehydration) compared to cattle directly sourced from ranches.

In addition to stress and pathogen transmission, administration of antimicrobials may alter the bacterial communities of the respiratory tract by reducing prevalence of susceptible bacteria. In contrast, colonization by resistant bacteria could potentially alter the prevalence and diversity of pathogens in the respiratory tract, with resistant strains outcompeting those that are susceptible to the antimicrobial administered. More recently, we reported high levels of resistance against tulathromycin and oxytetracycline in *M. haemolytica* and *P. multocida* (64.1 and 66.7%, respectively) isolated from feedlot cattle (12). In that study, it was not possible to evaluate the source of resistant bacteria as cattle were sampled at a single time point. While a subsequent study showed lower levels of antimicrobial-resistant BRD pathogens when calves were sampled on farms, compared to feedlot (13), there is still a paucity of information on the prevalence and resistance in BP-associated bacterial pathogens in cattle prior to feedlot arrival. Thus, whether farms or auction markets could potentially be a source of resistant bacteria, is currently unknown. The objective of this study was therefore to compare the prevalence and resistance of BP bacterial pathogens in beef cattle that were sent either directly to a feedlot or co-mingled at an auction market before transportation to a feedlot.

## 2. Materials and methods

### 2.1. Ethics statement

This study was performed in strict accordance with the recommendations of the Canadian Council of Animal Care (14). All procedures were reviewed and approved by the University of Calgary Veterinary Sciences Animal Care Committee (Protocol AC14-0192).

### 2.2. Animals

**Figure 1:**
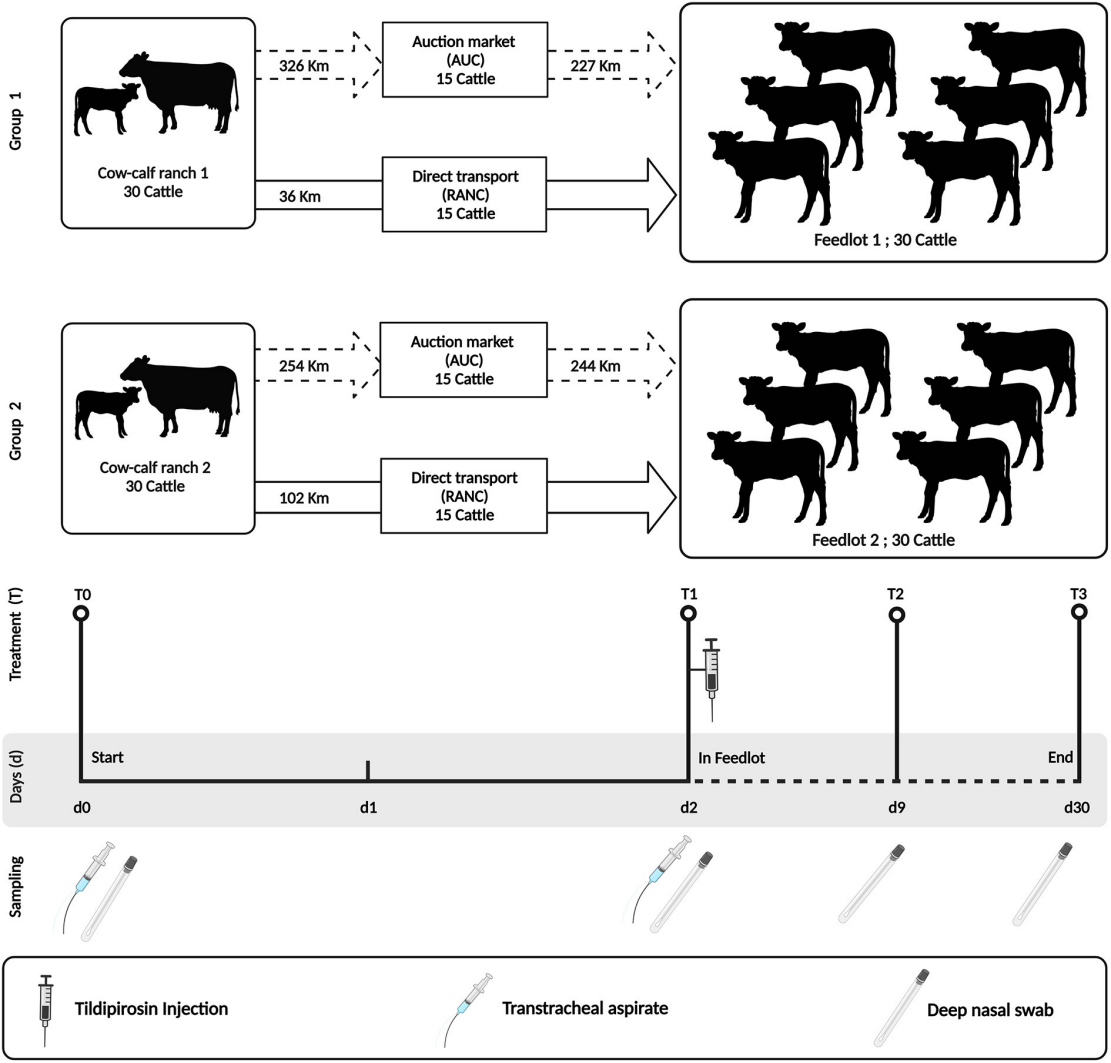
Study design indicating sampling time points and antimicrobial treatment. The study was performed using two groups of cattle (*N* = 30 per group). Group 1 cattle were placed in a high-capacity feedlot housing ~4,000 cattle (Feedlot-1). Group 2 cattle were placed in a low-capacity feedlot housing 600 cattle (Feedlot-2). Both groups were injected with tildipirosin at feedlot entry. All cattle were sampled similarly by deep nasal swab (DNS) and/or trans tracheal aspiration (TTA) at the indicated time points (T0–T3). This figure is created with BioRender.com.

Two groups of 30 angus-crossed beef heifers (*n* = 60) sourced from two cow-calf ranches were enrolled in the study and sampled from weaning to 28 days after entrance into a feedlot. The feedlots of each group of animals also differed, with Group 1 calves being placed in a high-capacity feedlot housing ~4,000 cattle (Feedlot-1) and Group 2 calves being placed in a low-capacity feedlot housing 600 cattle (Feedlot-2) (Figure 1).

At weaning on the cow-calf ranches (day 0 = T0), heifers were separated from their dams and their upper and lower respiratory tracts were sampled using a guarded deep nasopharyngeal swab (DNS) and trans-tracheal aspiration (TTA) kit, respectively. Thereafter, heifers were randomized into two groups of 15 animals and were transported either to a commercial auction market (AUCT) over a distance of 326 km (Group 1) and 254 km (Group 2), respectively. Heifers were kept at the auction market for 24 h in contact with other cattle, and then transported to respective feedlots over a distance of 227 km (Group 1) and 244 km (Group 2) on day 1. The other groups of 15 heifers were transported directly (RANC) from the cow-calf ranch to the feedlot over a distance of 36 km (Group 1) and 102 km (Group 2), respectively. During all steps of transportation, the heifers were not co-mingled with other cattle on the truck.

On day 2 (T1), all heifers (RANC and AUCT) were re-sampled by DNS and TTA. They also received subcutaneous injections of the macrolide tildipirosin (4 mg/kg, Zuprevo, Merck animal health, Kirkland, QC, Canada), vaccines against bovine rhinotracheitis virus, bovine viral diarrhea virus (types 1 and 2), bovine parainfluenza 3 virus, bovine respiratory syncytial virus, and *Mannheimia haemolytica* toxoid (Pyramid FP 5 + Presponse SQ, Boehringer Ingelheim, Burlington, ON, Canada), and clostridial disease and *Histophilus somni* (Vision 8 somnus, Merck animal health, Kirkland, QC, Canada) and were dewormed using topical moxidectin (Cydectin, Boehringer Ingelheim, Burlington, ON, Canada). Group 1 received two pulses of chlortetracycline at a dosage of 5 g per cattle per day from days 1–5 and days 8–12 (Aureomycin 220, Zoetis, Kirkland, QC, Canada), whereas Group 2 did not receive in-feed chlortetracycline.

Deep nasal swabs and TTA were also collected on day 9 (T2) and day 30 (T3). Heifers with visual BP signs (e.g., depression, nasal or ocular discharge, cough or dyspnea) and a rectal temperature ≥ 40.0°C were defined as a BP case and treated with enrofloxacin (12.5 mg/kg, Baytril 100, Bayer Inc., Mississauga, ON, Canada).

### 2.3. Sampling procedures

Deep nasal swab (DNS) and trans-tracheal aspirates (TTA) were collected at weaning (T0) and at on-arrival processing at the feedlot (T1) (Figure 1). Deep nasal swab sampling was then repeated on days 9 and 30 after arrival at the feedlot (T2 and T3, respectively).

Deep nasal swabs were collected as described (11) using long guarded swabs (27 cm) with a rayon bud (MW 124, Medical Wire & Equipment, Corsham, UK). Trans-tracheal aspirates were collected as described (7) using a 75-cm long catheter with an outside diameter of 2 mm (Centracath, Vygon, Ecouen, France). Immediately after sampling, deep nasal swabs were placed into sterile liquid Amies and refrigerated at 4°C. Fluid recovered from TTA (on average, 5–10 mL) was transferred into sterile plain tubes and also refrigerated at 4°C. Both DNS and TTA samples were shipped overnight to the Lethbridge Research and Development Center, Agriculture and Agri-Food Canada (AAFC) and processed within 12 h after arrival.

### 2.4. Pathogen isolation and identification

Upon receipt, the nasal swabs were removed from the transport medium and placed individually into 250 μl of brain heart infusion (BHI) broth and glycerol (60:40). For TTA, 250 μl of a sample was suspended in 250 μl of brain heart infusion (BHI) broth and glycerol (60:40). For bacterial isolation, the samples were vortexed and a 100-μl aliquot of sample suspension was used for culturing. The nasal swabs and the remaining amount of the TTA suspension were stored at −80°C.

For *M. haemolytica* and *P. multocida*, a 100-μL aliquot of the DNS and TTA suspensions were plated onto tryptic soy agar (TSA) containing 5% sheep blood and 15 μg/mL of bacitracin [to limit the growth of Gram-positive bacteria; (15)] and were incubated for 24 h at 37°C. For culturing of *H. somni*, 100 μL of the DNS and TTA suspension was plated onto TSA-blood plates without bacitracin and incubated for 48 h in a 10% CO_2_-enriched environment at 37°C. Up to three colonies from a plate displaying morphology indicative of *M. haemolytica* (white-gray, round, medium-sized, non-mucoid, exhibiting β-haemolysis), *P. multocida* (translucent, grayish in color, and mucoid in consistency), and *H. somni* (yellowish hue, haemolytic) were confirmed by PCR, as described previously (16). Positive isolates of *M. haemolytica, P. multocida*, and *H. somni* were then stored at −80°C for further characterization.

### 2.5. Antimicrobial susceptibility testing

For each bacterial species, one randomly selected isolate (when more than one isolate was banked) for nasal and tracheal samples from a single animal was analyzed to determine antimicrobial susceptibility. Antimicrobial susceptibility testing was performed by microdilution (Sensititre, Thermofisher Scientific, Nepean, ON, Canada) using a commercially available panel (BOPO6F custom bovine plates, TREK diagnostic systems, Cleveland, OH, USA) as described (17). Plates were automatically read using Vizion Digital MIC viewing (TREK diagnostics). Antimicrobials, range of concentrations tested, and minimum inhibitory concentrations (MIC) are listed in Supplementary Table S1 for *M. haemolytica*, Supplementary Table S2 for *P. multocida*, and Supplementary Table S3 for *H. somni*. Isolates were defined as resistant to antimicrobials according to MIC defined by the Clinical and Laboratory Standards Institute for ampicillin (≥0.25 μg/mL), ceftiofur (≥8 μg/mL), enrofloxacin (≥2 μg/mL), florfenicol (≥8 μg/mL), penicillin (≥1 μg/mL), oxytetracycline (≥8 μg/mL), tilmicosin (≥32 μg/mL, for *M. haemolytica*), and tulathromycin (≥64 μg/mL), [VET01S-Ed5, (18)]. CLSI breakpoints were not available for clindamycin, chlortetracycline, danofloxacin (for *H. somni*), gentamycin, tiamulin, tilmicosin (for *H. somni*), trimethoprim/sulfamethoxazole, tylosin, and neomycin. Therefore, susceptibility designations were not assigned for these compounds, with the exception of neomycin. For neomycin, resistance was defined as an MIC ≥ 32 μg/ml according to Klima et al. (17). Reference strains *Staphylococcus aureus* ATCC 29213, *Escherichia coli* ATCC 25922, and *Enterococcus faecalis* ATCC 29212 served as quality controls.

### 2.6. Pulsed field gel electrophoresis

Isolates were typed by PFGE according to the standardized protocol for Molecular subtyping from Pulsenet (19) and previously described (20) using a CHEF DR II electrophoresis unit (Bio-Rad Laboratories, Mississauga, ON). Restriction digestions were completed using SalI for *M. haemolytica*, ApaI for *P. multocida*, and SacII for *H. somni*. Electrophoresis gels were run at 6 V/cm and electrophoresis conditions were a 4.0 s initial switch time and 40 s final switch time for 20 h at 12°C. Resulting patterns were analyzed using Bionumerics Version 7.1 software (Applied Maths Inc., Austin, TX). Dendrogram and minimum spanning tree (MST) analysis were based on similarity matrices generated from UPGMA clustering of Dice coefficient values with 1.0% tolerance and 0.5% optimization. Bin size for MST was set to 3.0% and complexes were generated with a maximum neighbor distance of 2. Isolates with ≥90% similarity according to BioNumerics analysis were grouped as a pulsotype.

### 2.7. Statistical analysis

#### 2.7.1. Pathogen isolation

Isolation rates of pathogens from DNS and TTA sample types between groups (AUC and RANC) at T0 were compared using chi-square tests in Microsoft Excel^®^. Effects of auction market on the isolation rates of pathogens from DNS sample types were assessed using a mixed effects logistic regression model, performed in R (R Core Team, Vienna, Austria). Variables included in this model were AUC and RANC cattle and different time points (T1, 2, 3). Deep nasal swabs and TTA were used as the experimental unit. Effects of auction market on the isolation rates of pathogens from TTA sample types were compared using a chi-square test in Microsoft Excel^®^. RANC and AUC samples were analyzed separately at each time point and in each group. For all analyses, a *P-*value of 0.05 was considered significant.

## 3. Results

### 3.1. Prevalence data

**Figure 2:**
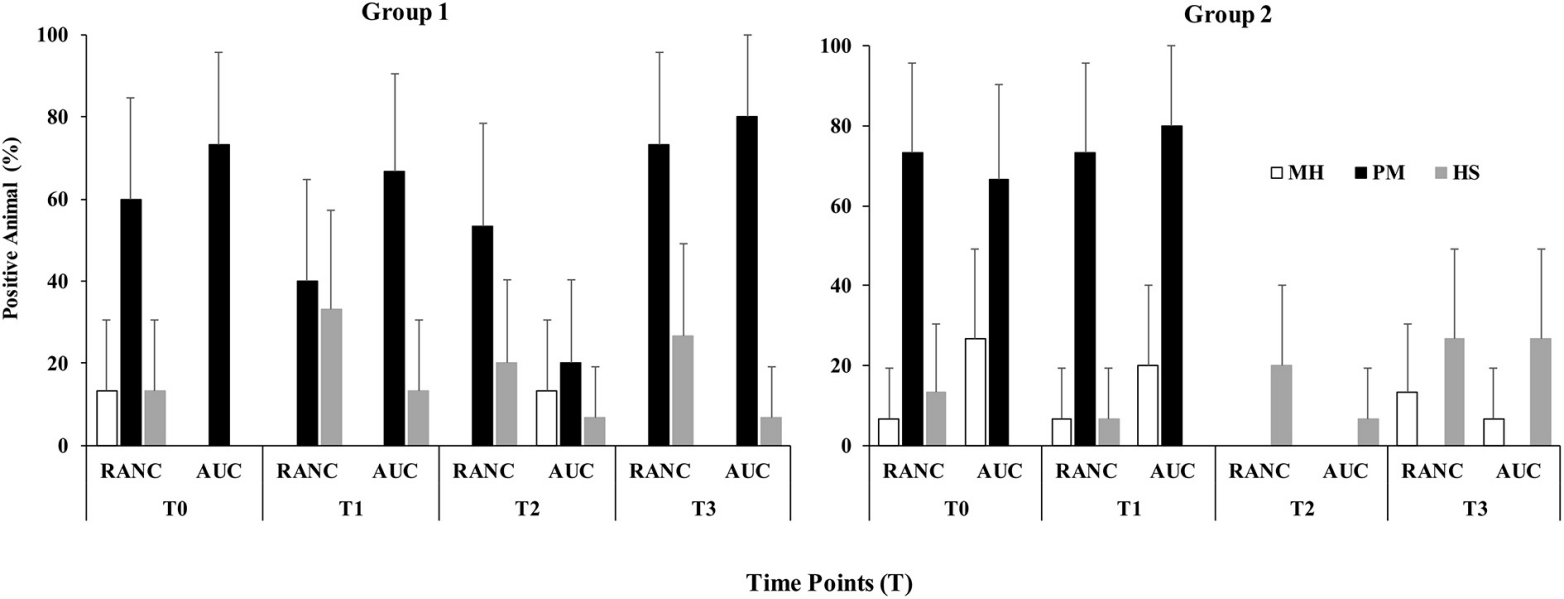
Prevalence of *M. haemolytica* (MH), *P. multocida* (PM), and *H. somni* (HS) in DNS samples from auction-derived (AUC) and ranch-direct (RANC) cattle for Group 1 and Group 2. Isolation rates of *M. haemolytica* (*P* > 0.05), *P. multocida* (*P* > 0.05), and *H. somni* (*P* > 0.05) did not differ between AUC and RANC at T1, T2, and T3 in both feedlots. The error bars represent 95% confidence interval.

**Figure 3:**
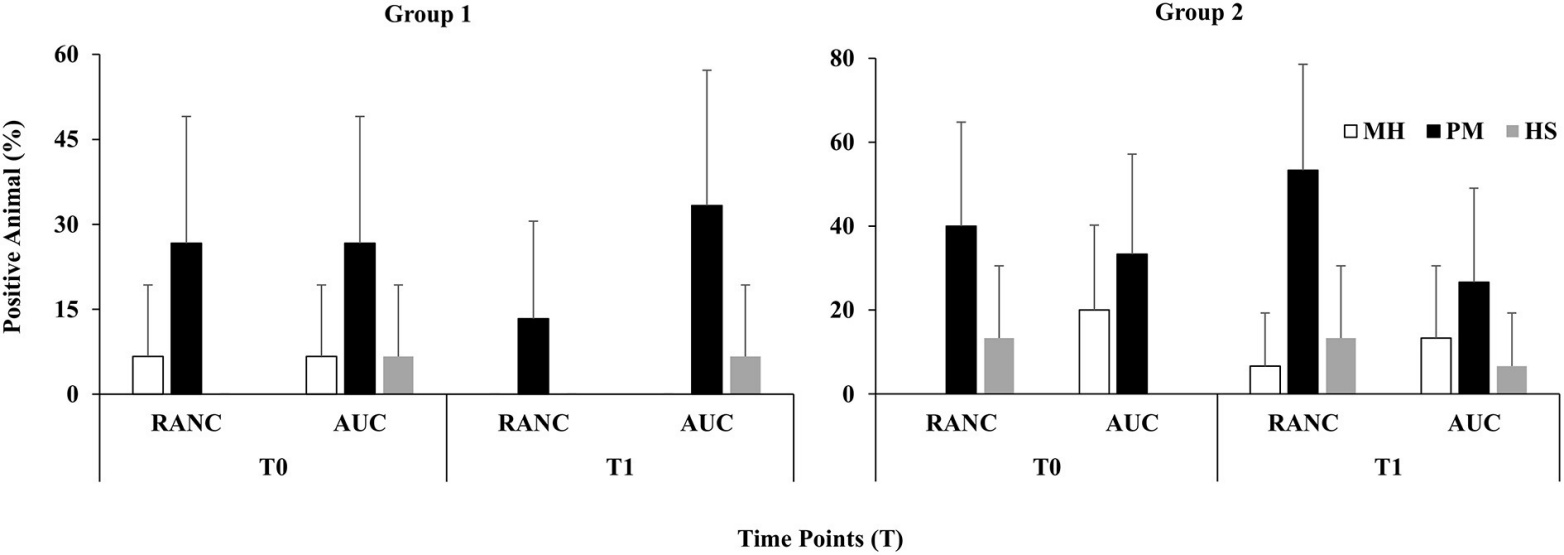
Prevalence of *M. haemolytica* (MH), *P. multocida* (PM), and *H. somni* (HS) in TTA samples from auction-derived (AUC) and ranch-direct (RANC) cattle for Group 1 and Group 2. Isolation rates of *M. haemolytica* (*P* > 0.05), *P. multocida* (*P* > 0.05), and *H. somni* (*P* > 0.05) did not differ between AUC and RANC at T0 and T1 in both feedlots. The error bars represent 95% confidence interval.

A schematic of the study design is shown in Figure 1. Across all time points and respiratory sample types, *P. multocida* was the most frequently isolated pathogen from RANC and AUC cattle in Group 1 (62.2%) and Group 2 (40.0%) compared to *M. haemolytica* [Group 1 (5.0%), Group 2 (12.5%)] or *H. somni* [Group 1 (16.0%), Group 2 (15.8%)]. No difference in prevalence of pathogens isolated from DNS (*P* > 0.05) or TTA (*P* > 0.05) was observed between AUC and RANC at T0 in both groups. Isolation rates of *P. multocida* (*P* > 0.05), *M. haemolytica* (*P* > 0.05), and *H. somni* (*P* > 0.05) from DNS samples did not differ between AUC and RANC at T1, T2, and T3 in both groups (Figure 2). Similarly, isolation rates of *P. multocida* (*P* > 0.05), *M. haemolytica* (*P* > 0.05), and *H. somni* (*P* > 0.05) from TTA samples did not differ between the AUC and RANC groups at T0 and T1 in both groups (Figure 3). Prevalence of *P. multocida, M. haemolytica* and *H. somni* isolated from TTA samples did not change from the cow-calf ranch to arrival at the feedlot (between T0 and T1) (*P* > 0.05) in both groups (Figure 3). In Group 2 cattle, the prevalence of *P. multocida* isolated from DNS decreased in RANC and AUC cattle from T0 (73.3 and 66.7%, respectively) and T1 (73.3 and 80.0%, respectively) to time point T2 (0%) and T3 (0%) (Figure 2).

### 3.2. Pulsed-field gel electrophoresis and antimicrobial susceptibility

Genetic diversity of *M. haemolytica, P. multocida*, and *H. somni* did not appear to relate to whether cattle were direct or auction-derived, as most pulsogroups were identified in isolates from AUC and RANC cattle (Table 1). In addition, there were no strains that appeared specific to only the trachea, as most LRT isolates were from a pulsogroup that also included bacteria isolated from the URT. Three pulsogroups were observed for *M. haemolytica* and two for *H. somni* in Group 2 calves whereas one pulsogroup was observed for *M. haemolytica* and two for *H. somni* in Group 1 calves. The same pulsotype of *H. somni* was isolated from one animal in the AUC group and three cattle in the RANC group at T3 in Group 1. A total of 13 *M. haemolytica* (Group 1, *N* = 5; Group 2, *N* = 8) and 9 *H. somni* (Group 1, *N* = 5; Group 2, *N* = 4) were unique and did not share 90% genetic relatedness to any other isolates.

**Table 1:** Characterization of *Pasteurella multocida (PM), Mannheimia haemolytica (MH)*, and *Histophilus somni (HS)* isolates from cattle sampled by deep nasal swab (N) or trans-tracheal aspiration (T).

**Species**	**Pulso-group^c^**	**Cattle group**	**Auction market** ^**a**^				**Direct feedlot** ^**a**^				**Resistance phenotype^e^**
			**Sample type (no. of isolates)**				**Sample type (no. of isolates)**				
			**T0^b^**	**T1**	**T2**	**T3**	**T0**	**T1**	**T2**	**T3**	
PM	1	2	N(3), T(1)	N(1), T(1)			N(2), T(2)	N(2), T(2)			
	2	2	N(1)				N(1)	N(1)			
	3	2					N(1), T(1)	N(1), T(1)			
	4	2		N(1)				N(1)			
	5	2	T(1)	N(1)							
	6	1		N(1)			N(1)	N(1)			
	7	1	N(3), T(3)	N(5), T(2)			N(2), T(3)	N(4), T(5)			
	8	1		N(1)			N(3)				
	9	1	T(1)	N(1)							
	10^e^	1			N(3)	N(12)			N(8)	N(11)	OXY-TIL-TUL
	11	1	N(1)				N(1), T(1)				
	12	2	N(1)				N(1)				
	13^e^	2		T(1)			N(1)				CEF-PEN
	14	2	N(1)	N(1)				N(1)			
	15	2	N(2), T(4)	N(2), T(1)			N(3), T(1)	N(4), T(3)			
	16	2	N(1)	N(1)							
	17	2					T(1)	N(1), T(1)			
	18	2						N(1), T(1)			
	Other^d^	1	N(7)	N(2)			N(2)	N(1), T(1)			
	Other^d^	2		N(3)			N(2), T(1)	N(2)			
MH	1^e^	1				N(1)				N(1)	OXY-TIL
	2	2	N(3)	N(1)			N(1)				
	3	2	T(1)	T(1)				T(1)			
	4	2	T(1)	T(1)							
	Other^d^	1	T(1)	N(1)			N(2), T(1)				
	Other^d^	2	N(1), T(1)	N(1), T(1)		N(1)		N(2)		N(1)	
HS^f^	1^e^	1				N(1)				N(3)	OXY
	2	2						N(2)			
	3	1,2					N(1), T(1)	N(1)	N(1)	N(1)	
	Other^d^	1		N(2)	T(1)			T(1)		N(1)	
	Other^d^	2	T(1)	T(1)			T(1)	N(1)			

^a^Cattle were transported from the auction market to the feedlot or transported directly to the feedlot.

^b^Timepoints were T0: cow-calf ranch; T1: day 2; T2: day 9; T3: day 30.

^c^Pulsogroups (90% similarity) were derived from dendrograms created using UPGMA clustering of dice coefficient values.

^d^Other indicates the isolates of cattle that did not have >90% similarity to any other isolate of the species.

^e^Resistance phenotypes (shaded) were determined by broth dilution and defined as resistant according to CLSI guidelines. OXY, oxytetracycline; TIL, tilmicosin, TUL, tulathromycin; CEF, ceftiofur; PEN, penicillin.

^f^Only 20 out of 40 *Histophilus somni* isolates could be typed.

**Figure 4:**
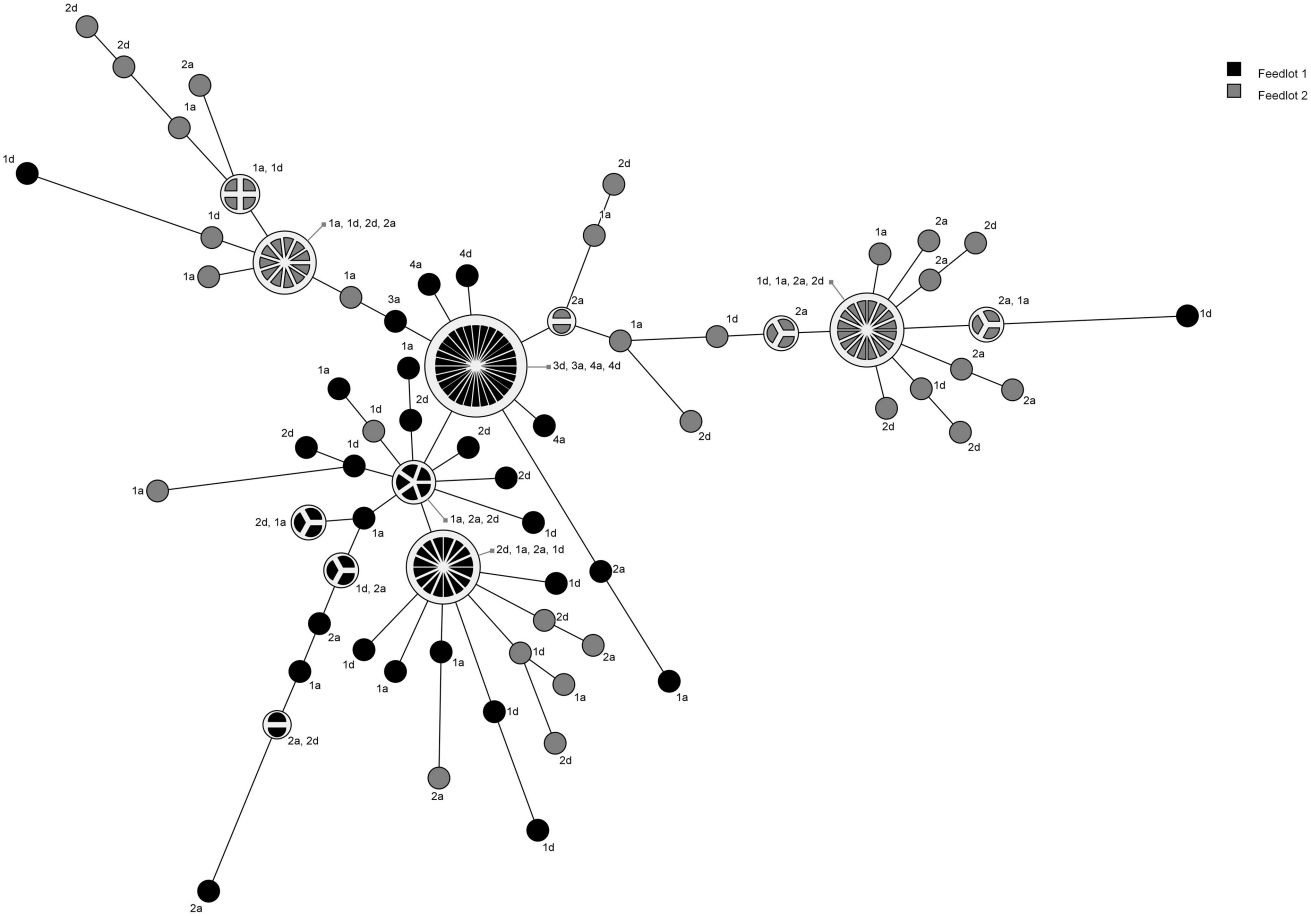
Minimum spanning tree based on a similarity matrix generated from pulsed-field gel elctrophoresis profiles of *P. multocida* isolates collected from cattle in Groups 1 and 2. Distance between isolates indicates the similarity. Shading indicates group of animals the bacteria were isolated from (black = Group 1, gray = Group 2). Numbers refer to sampling times (1 = day 0, 2 = day 2, 3 = day 9, 4 = day 30). Letters represent the treatment (D, direct transport; A, auction).

**Figure 5:**
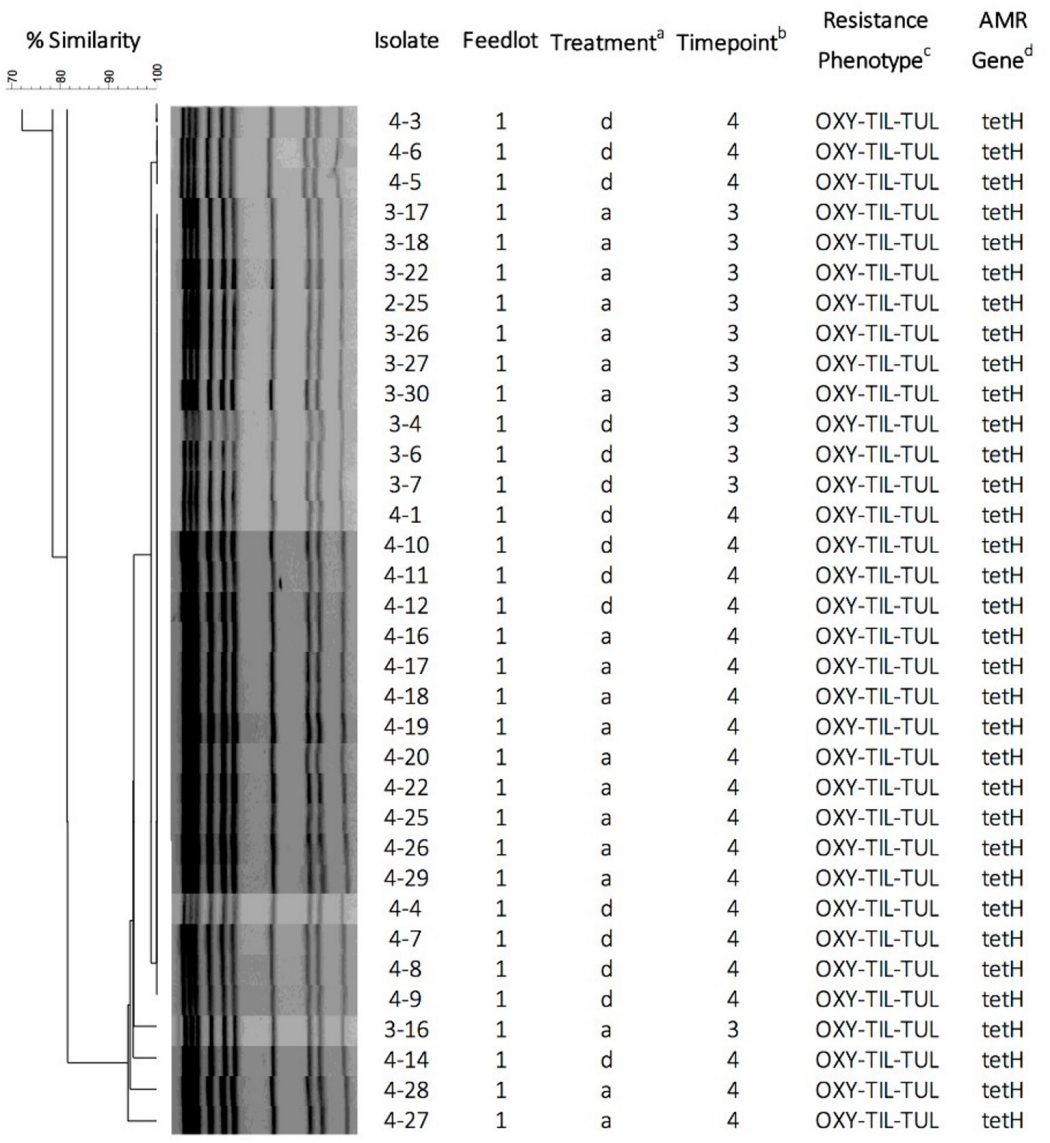
Pulsed-field gel electrophoresis dendrogram, cluster analysis, and antimicrobial susceptibility for 34 *P. multocida* isolates representing pulsogroup 10 (90% similarity threshold cutoff value). *P. multocida* were digested with ApaI. The dendrogram was created by UPGMA cluster analysis using Dice coefficients of similarity with optimization and tolerance settings of 0.5 and 1.0%, respectively. ^a^Treatment: d, calves directly transported from cow-calf ranch to feedlot; a, calves transported to and co-mingled at auction markets before placement in a feedlot; ^b^Sampling timepoints: 3 = day 9; 4 = day 30; ^c^Phenotypic resistance against antimicrobials OXY (oxytetracycline), TIL (tilmicosin), and TUL (tulathromycin). ^d^Antimicrobial resistance genes detected by PCR.

A total of 18 pulsogroups (Group 1, *N* = 6; Group 2, *N* = 12) were observed for *P. multocida*, whereas 21 isolates were unique and did not share 90% genetic relatedness with other strains (Group 1, *N* = 13; Group 2, *N* = 8). Pulsogroup 10 was most frequently isolated (*N* = 34) and all bacteria within this group were from time points T2 and T3 in Group 1, whereas no other pulsogroups were observed after T1. *Pasteurella multocida* was not isolated from cattle in Group 2 after T1. Thus, high genetic diversity with up to 12 pulsogroups in Group 2 and six in Group 1 was observed prior to feedlot placement and at arrival at the feedlot in both auction-derived and ranch-direct cattle. The level of genetic diversity decreased with days on feed at the feedlot, as illustrated by a reduction in pulsogroups after T1. In support of this, when pulsotypes were analyzed using a similarity matrix and presented as a minimum spanning tree, it was clear that only one dominant clade was present at time points T2 and T3, whereas multiple clades were observed in Group 1 and 2 animals at T0 and T1 (Figure 4). The genetic relatedness of *P. multocida* pulsogroup 10 was high, with most isolates in this group having a common PFGE restriction pattern and indicating a clonal origin (100% related; Figure 5).

None of the bacteria isolated at time points T0 and T1 were resistant to any of the antimicrobials tested (Table 1). Only one pulsogroup of *M. haemolytica*, containing two isolates, were resistant to oxytetracycline and tilmicosin. These isolates were from Group 1 AUC and RANC cattle and were recovered at T3. Similarly, only one pulsogroup of *H. somni*, containing four isolates, was resistant to oxytetracycline. These *H. somni* were also recovered from both AUC and RANC cattle in Group 1, at T3. Two isolates of *P. multocida* (pulsogroup 13) isolated from Group 2 cattle were resistant to ceftiofur and penicillin and were recovered at T0 and T1 from RANC and AUC cattle, respectively. All other isolates of *P. multocida* from T2 and T3 (Group 1 cattle) were within pulsogroup 10 and shared an oxytetracycline-tilmicosin-tulathromycin multidrug resistant phenotype. Pulsogroup 10 *P. multocida* isolates (*N* = 34) were cultured from 24 cattle in Group 1. An attempt was made to screen for genes conferring resistance to oxytetracycline [*tet*(H)] and macrolides [*msr*(E), *mph*(E), *erm*(42), *erm*(A), *erm*(B), *erm*(C), *erm*(F), *erm*(T) and *erm*(X)] in resistant isolates. All bacteria displaying oxytetracycline resistance harbored *tet*(H), whereas none of the macrolide resistance genes were detected in any of the macrolide-resistant isolates.

### 3.3. Health data

Nine of the 30 heifers (30%) in Group 1 were diagnosed and treated for BP at d 9; five heifers within RANC group and four heifers within AUC cattle. In Group 2, three heifers (10%) were diagnosed with BP and treated; one within RANC at d 2 and two within AUC cattle at d 2 and 8 of the study. No other diseases were observed.

No differences were observed between heifers that remained healthy and those diagnosed with BP in pathogen isolation rates of nasopharyngeal samples on any sampling day.

## 4. Discussion

The hypothesis that auction market cattle are at high risk for BP at entrance into feedlots because they acquire pathogens during the process of co-mingling at auction markets was not supported in the present study. While some new strains for each of the pathogens were identified at T1 (feedlot entry), this was the case for both AUC and RANC cattle, and none of these strains persisted to T2 and T3 (Table 1). Thus, specific instances of a strain presenting in AUC cattle after co-mingling was not observed. While caution should be used in interpreting these data, due to the small sample size, this study suggests that previous reports indicating greater susceptibility to BP in auction market cattle may have been due to a multitude of stressors from the marketing process, such as sorting and loading, transportation, and co-mingling of cattle. These procedures result in increased stress periods which compromise the local and systemic immune defenses predisposing cattle to respiratory disease (21). It should be noted that viral agents were not investigated in the present study, and whether transfer occurs during co-mingling at the auction markets could not be determined.

Results of this study demonstrated that high heterogeneity of BP pathogens was present at the first two time points, prior to long-term feedlot placement. The level of genetic diversity decreased by days 9 and 30, and for *P. multocida*, and no isolates were recovered at the last two time points in Group 2 animals. The decrease was likely due to antimicrobial administration. Although limited information on the active concentrations of antibiotics in the upper respiratory tract of treated cattle exist, oxytetracycline has been shown to achieve therapeutic concentrations in oral fluid (22) and nasal secretions (23) of pigs after intramuscular administration. It is likely that some secretion of tildipirosin and chlortetracycline occurred, reducing the pathogens in the nasopharynx. However, this decline in genetic variation does not correlate with the findings reported by Klima et al. (24) where there was a greater extent of genetic diversity observed among isolates of *M. haemolytica* at exit of the feedlot. In that study however, cattle were administered metaphylactic doses of oxytetracycline or tulathromycin, and the sampling time was twice as long as our study, perhaps accounting for these differences observed.

Interestingly, none of the bacteria from time points T0 and T1 were resistant to any antibiotics tested. This would suggest that antimicrobial use in feedlots selects for resistant pathogens, though it should be noted that the cow-calf-ranch use of antimicrobials for the two groups of cattle was unknown, and it cannot be ruled out that antimicrobial administration of calves prior to feedlot placement does not also select for resistant bacteria. Except for one isolate of *P. multocida* from a Group 2 calf (ceftiofur/penicillin-resistant phenotype), all resistant phenotypes observed corresponded to the classes of antimicrobials administered (i.e., tetracyclines and macrolides). It is interesting to note that no other resistant bacteria were isolated from Group 2 cattle. The feedlot that Group 1 calves were placed in was larger but also administered therapeutic doses of chlortetracycline. Thus, it cannot be ascertained as to whether feedlot size or in-feed chlortetracycline resulted in the differences observed between Group 1 and 2 cattle. The fact that a common pulsogroup of *P. multocida* colonized the majority of AUC and RANC Group 1 cattle after feedlot placement supports that antimicrobial treatment selected for a resistant strain in Group 1. As this strain was not observed prior to time point T2, it is possible that it was endemic to the feedlot. Transmission of *P. multocida Type B:2* between buffalo calves was reported before (25). Furthermore, evidence of clonal spread and transmission of *M. haemolytica* within the feedlot has been previously observed (7, 17, 26). This highlights the importance of monitoring resistance in feedlots and making strong efforts to enlist management practices that do not promote the spread of resistant pathogens to new arrivals or between sick and healthy calves.

The antimicrobial resistant phenotype of the dominant pulsogroup (pulsogroup 10), which was found in Group 1 cattle, conferred macrolide and tetracycline resistance. Although tildipirosin was not available to test on the antimicrobial panel used in our study, it is likely that this antimicrobial can select for resistance to tulathromycin and tilmicosin, which were tested. In a previous longitudinal study (2007–2012) monitoring bacterial resistance, a lack of tulathromycin resistance was observed in cattle sampled at entry and at 60 DOF (27). However, several studies have now shown widespread macrolide resistance in *P. multocida* and *M. haemolytica* (28–30), indicating that rates of resistance are increasing. The dissemination of a single clone from 34 AUC and RANC cattle within 3 weeks of sampling indicates a high horizontal transmission rate of resistant isolates within the feedlot. That all of these isolates displayed oxytetracycline and tilmicosin/tulathromycin resistance, indicated that genes conferring their resistance may be harbored on a common element (16). It is interesting that previously identified macrolide resistance genes could not be detected in any of the isolates. This would suggest that an unknown mechanism is conferring macrolide resistance in these isolates.

*Pasteurella multocida* was identified as the predominant pathogen in the current study, which is consistent with results reported previously (12, 31). However, this finding suggests that this pathogen is emerging at higher frequencies and gaining dominance over *M. haemolytica*, which was considered the most prevalent pathogen causing BP over the last decades (32, 33). Reasons could be that currently used vaccines are more effective against *M. haemolytica* or current antimicrobial treatments are more effective for reduction of *M. haemolytica* (34). It seems that *P. multocida* is more likely to develop antimicrobial resistance and it is also possible that acquisition of antibiotic resistance genes by genetic drift could promote more resilient populations (35), as was suggested by our study. The antimicrobial usage history (metaphylactic, therapeutic, subtherapeutic) in cattle is likely correlated to newly acquired antimicrobial resistance strains present in feedlots and also likely contributes to reducing bacterial diversity.

## 5. Conclusions

Although the sample size was limited, we concluded that for the calves enrolled in this study, transportation to and co-mingling at an auction market for 24 h did not result in acquisition of major bacterial respiratory pathogens (e.g., *M. haemolytica, P. multocida*, and *H. somni)*. Furthermore, horizontal transmission of a multi-resistant strain of *P. multocida* among calves can occur in the feedlot. Clonal spread of resistant bacteria was likely due to the selective pressures imposed by feedlot antimicrobial use and encoded resistance by the bacteria.

## Data availability statement

The original contributions presented in the study are included in the article/Supplementary material, further inquiries can be directed to the corresponding author.

## Ethics statement

The animal study was reviewed and approved by University of Calgary Veterinary Sciences Animal Care Committee (Protocol AC14-0192).

## Author contributions

CH was involved in study design, sample collection, laboratory work, and wrote the initial manuscript. ET and TA were involved in concept development, study design, and manuscript preparation. MU was involved in figure preparation and assisted with laboratory work. LG contributed in figure and manuscript preparation. All authors read and approved the final manuscript.
